# Uncertainty Estimates of Purity Measurements Based on Current Information: Toward a “Live Validation” of Purity Methods

**DOI:** 10.1007/s11095-012-0836-z

**Published:** 2012-08-15

**Authors:** Izydor Apostol, Drew Kelner, Xinzhao Grace Jiang, Gang Huang, Jette Wypych, Xin Zhang, Jessica Gastwirt, Kenneth Chen, Szilan Fodor, Suminda Hapuarachchi, Dave Meriage, Frank Ye, Leszek Poppe, Wojciech Szpankowski

**Affiliations:** 1 Amgen, Analytical Sciences, Thousand Oaks, California 91320 USA; 2 Amgen, Global Analytical Sciences, Longmont, Colorado 80503 USA; 3 Amgen, Product Attribute Sciences, Thousand Oaks, California 91320 USA; 4 Amgen, Clinical Quality, Thousand Oaks, California 91320 USA; 5 Amgen, Corporate Quality Engineering, Thousand Oaks, California 91320 USA; 6 Amgen, Molecular Structure, Thousand Oaks, California 91320 USA; 7 Purdue University, Department of Computer Science, West Lafayette, Indiana 47907 USA

**Keywords:** live validation, method precision, purity methods, uncertainty, UBCI

## Abstract

**Purpose:**

To predict precision and other performance characteristics of chromatographic purity methods, which represent the most widely used form of analysis in the biopharmaceutical industry.

**Methods:**

We have conducted a comprehensive survey of purity methods, and show that all performance characteristics fall within narrow measurement ranges. This observation was used to develop a model called Uncertainty Based on Current Information (UBCI), which expresses these performance characteristics as a function of the signal and noise levels, hardware specifications, and software settings.

**Results:**

We applied the UCBI model to assess the uncertainty of purity measurements, and compared the results to those from conventional qualification. We demonstrated that the UBCI model is suitable to dynamically assess method performance characteristics, based on information extracted from individual chromatograms.

**Conclusions:**

The model provides an opportunity for streamlining qualification and validation studies by implementing a “live validation” of test results utilizing UBCI as a concurrent assessment of measurement uncertainty. Therefore, UBCI can potentially mitigate the challenges associated with laborious conventional method validation and facilitates the introduction of more advanced analytical technologies during the method lifecycle.

**Electronic supplementary material:**

The online version of this article (doi:10.1007/s11095-012-0836-z) contains supplementary material, which is available to authorized users.

## INTRODUCTION

The safety and efficacy of biopharmaceuticals is controlled by measurements of their quality attributes. To measure these attributes for protein pharmaceuticals, a set of analytical methods are developed that have to meet the requirements specified by the International Conference on Harmonization (ICH) guideline Q6b, “Specifications: Test procedure and acceptance criteria for biotechnological/biological products” (1). The largest category of methods is made up of purity (and impurity) methods used to determine the relative abundance (normalized concentration) of defined attributes. The term purity and relative abundance will be used interchangeably throughout the manuscript. Typically, these methods involve chromatographic and electrophoretic separations. Because of the molecular complexity of biopharmaceuticals, the purity of protein drugs is generally assessed by a larger number of analytical methods relative to those used for purity determination of small molecule drugs (2). Therefore, the measurement of purity and the assessment of uncertainty of the test results play important roles for the biotechnology industry in controlling product quality. The appraisal of the uncertainty of results is performed during formal method validation.

Since the necessity of method validation has been reinforced by a variety of national and international regulations (3–6) industry practitioners fully understand the importance of method validation as a means of ensuring the scientific validity of results. However, existing guidance is subject to user interpretation leading to the potential for confusion when it comes to carrying out the validation exercise. However, numerous articles have been published to provide scientific justification for the importance of this activity, and to alleviate the vagueness of these regulations (7–13). The most frequently referenced publication is the ICH guideline Q2R1, “Validation of analytical methods: text and methodology” (6). This document covers validation activities targeted at product registration; hence, this guidance is specifically applicable to commercial products at the time of the product development lifecycle when the marketing application is submitted to the regulatory authorities. As a matter of industry practice, many, if not most, practitioners carry out Q2R1 compliant method validation activities to enable use of the validated methods for the testing of the process validation lots. While there is no specific guidance on method validation for earlier stages of product development, regulatory expectations and industry practices have evolved to provide assurance of acceptable method performance in earlier development stages. The generally accepted term for these pre-commercial activities is method qualification (9,14), but the scope of the qualification, timing with respect to the various stages of product development, and the relationship of qualification to validation activities has not been consistently delineated or practiced. In fact, method qualification is not mentioned in available guidelines, so it is difficult to define its scope and significance, although it is clear that both qualification and validation activities should be carried out by qualified (i.e.; properly trained) personnel using qualified instrumentation that has been shown to be fit for its intended purpose.

As a matter of industry practice, most practitioners carry out qualification activities to enable release and stability testing of GMP lots used in the clinic at all times prior to the availability of fully validated methods around the time of the process validation lots, as described above. Typical validation/qualification studies include evaluation of the following performance characteristics: precision (repeatability and intermediate precision), specificity, accuracy, detection limit (DL), quantification limit (QL), linearity and range. In many cases, numerous sample types are subject to these studies. Therefore, qualification and/or validation studies are very labor intensive, and often can create a significant bottleneck in the analytical lifecycle, which, in turn, significantly contributes to the cost of development and the cost of quality.

The uncertainty of results is a parameter that describes a range within which the measured value is expected to lie (15). Intuitively, we associate this parameter with precision. Therefore, method precision has been viewed as the most important performance characteristic relevant to establishing specifications (16), method transfer requirements (17), method robustness (12), assessment of process variability, etc. (18,19).Typically, method precision has been assessed from replicate analyses of the same sample. The work of Hayashi and Matsuda on FUMAI theory (20–25) demonstrated that the precision of chromatographic methods can be predicted from noise and the height and width of the signal (peak). However, due to the complexity associated with the required Fourier transformation of chromatograms and the parameterization of the power spectrum called for in implementation of this theoretical construct to the determination of precision, the FUMAI theory approach has not been widely applied. Similarly, attempts have been made to predict DL and QL based on chromatographic information (26–28); however, other performance characteristics have not been addressed.

In this manuscript, we present a new approach to assessing the uncertainty (performance characteristics) of purity analysis using a more holistic approach which we call Uncertainty Based on Current Information (UBCI). The model allows for real-time assessment of all performance characteristics using the results of the specific separation of interest. A fundamental, underlying principle of this approach recognizes that the execution of a purity method is always associated with specific circumstances; therefore, uncertainty about generated results needs to account for both the operational conditions of the method and the hardware. It is important to note that historical qualification/validation approaches do not take this fundamental principle into account, such that performance drift may occur over time due to hardware differences and even due to differences in analyst skill levels, such that the uncertainty of results obtained early in the product lifecycle may not be fully applicable to results obtained later following product commercialization.

We will show that signal and noise levels, instrument settings and software settings can be linked directly to all method performance characteristics. Such simplification/generalization makes it easy to implement this procedure in a daily operation, and can provide a valuable live assessment of uncertainty instead of extrapolating uncertainty from historical qualification/validation studies. As noted above, application of historical validation data always begs a question about the relevance of these data to the current experimental situation, and sometimes requires investigation, which can delay the approval of results. This new approach, therefore, has the capability of providing not only simplicity, but also a greater level of assessment of the data validity relative to current practices. We view this as an important step toward ‘live validation’ of purity methods.

## MATERIALS AND METHODS

### Qualification for Purity Methods

To demonstrate the suitability of the methods for their intended purposes, Amgen has established an internal guideline to govern and streamline the method qualification process.

System suitability: Each set of experiments has be preceded with three and bracketed (ended) with two injections of the reference material to satisfy requirements of system suitability (4). Parameters of system suitability included resolution and reproducibility requirement for the reference standard material used by the method. Blank injections were added occasionally.

A series of experiments was designed to evaluate the method performance against qualification target expectations. The performance characteristics that were addressed for purity methods included specificity, linearity, precision, accuracy, range, DL, and QL. Specificity was determined by comparing the sample and sample matrix blank to assure that no significant interference from the sample matrix with reported peaks was present. In addition, carryover and recovery were calculated to measure the specificity of the method.

% carryover was calculated using the equation below:

1 $$ Carryover(\% ) = \frac{{{B_S} - {B_B}}}{{{A_S}}} \times 100\% $$

where B_s_ is the total integrated peak area in blank injected after sample, B_B_ is the total integrated peak area in blank injected before sample, and A_S_ is the total integrated peak area in sample.

Protein recovery and % recovery were calculated using the equations below (29,30):

2 $$ Protein \_ Recovered(\mu g) = \frac{{A \times \frac{F}{{60}} }}{{\varepsilon \times l}} $$

where *A* is the total peak area in mAU*sec, *F* is the flow rate in mL*min^−1^, *ε* is the extinction coefficient at detection wavelength, (or 280 nm) in AU*mL*mg^−1^*cm^−1^, and *l* is the path length of flow cell in cm.

3 $$ {\text{Recovery}} = \left( {{\text{Protein}}\,{\text{recovered}}/{\text{Protein}}\,{\text{injected}}} \right) \times {1}00\% $$

The initial theoretical extinction coefficient for proteins was calculated from the amino acid composition (31). An experimental extinction coefficient was also determined using amino acid analysis. If the experimental value agrees within 10% of the theoretical value then, theoretical extinction coefficient was used throughout the studies. Otherwise, experimental extinction coefficient was used. In all cases, the protein samples were highly purified, with less than 100 ppm of host cell protein impurities present.

Linearity of the method was established by calculating the coefficient of determination (R^2^) to correlate the total peak area with protein load from 50% to 150% of target load. Linearity of minor species was evaluated by performing a blending study with minor species-enriched samples to show that the minor species behaved similarly as the main product. Precision was assessed for both repeatability and intermediate precision. Repeatability was determined by calculating the % Relative Standard Deviation (RSD) of 3 determinations of the 5 concentrations within the linear range.. Intermediate precision was evaluated by calculating the % RSD of 3 determinations of target load from 5 different experiments, accounting for differences from the day of experiment, analyst, equipment and column/capillary lot. Accuracy was expressed as 100 × (1– slope), in which slope was obtained by regression analysis plotting % main peak *vs.* % target load. Range was the low and high end of protein concentrations of the linearity study. Limit of detection (DL) and limit of quantitation (QL) were estimated using the statistical approach described previously (32) Execution of all qualification experiments required at a minimum 101 injections, which can be completed in five sequences.

### Purity Assessment by SE-HPLC, CEx-HPLC, and rCE-SDS, Peptide Map and Glycan Map

SE-HPLC (Size Exclusion HPLC): the protein sample was loaded SE-HPLC column (TSK-GEL G3000SWXL, 7.8 mm × 300 mm, 5 μm particle size, Tosoh Bioscience), separated isocratically with phosphate buffer containing sodium chloride salt, and the eluent was monitored by UV absorbance. Samples were injected neat without prior dilution.

CEx-HPLC (Cation Exchange HPLC): the charge variants of mAbs were separated on a cation exchange column (Dionex WCX-10, 4.0 mm × 250 mm, 10 μm particle size, Dionex) by increasing the salt concentration (hereby the ionic strength of mobile phase). The eluent was monitored by measuring the UV absorbance. For analysis of mAbs, mobile phase A was based on a MES buffer, mobile phase B contained MES buffer with NaCl salt. Samples were injected neat without prior dilution.

rCE-SDS (reduced Capillary Electrophoresis in SDS): rCE-SDS was performed on a Beckman Coulter ProteomeLab PA800 CE. The protein was reduced with β-mercaptoethanol. The reduced protein species were bound to SDS, an anionic detergent, and electrokinetically injected into a bare fused silica capillary. The SDS coated protein species were separated with SDS gel buffer (15 kV for 30 min), and detected with UV at 220 nm by a photodiode array (PDA) detector.

Peptide maps: Peptide mapping is an analytical method in which the test protein sample is first digested into peptides by a site-specific endoproteinase. The peptides are then resolved by reversed phase HPLC to generate a pattern of peaks. Samples were digested at 37°C for 16 h with a 1:100 enzyme to substrate ratio using Lys-C endoproteinase. 50 μg of digested protein was then separated using a 0.1% TFA in water/acetonitrile gradient using a C8 reversed-phase HPLC column. During method development, in line mass spectrometer was used to confirm identity of peptides (33).

Glycan map:. The glycans on the antibody were released by adding 1 μL of PNGase F to every 100 μg of protein. The released glycans were labeled by adding 10 μL labeling reagent (0.35M 2AB, 1 M NaCNBH3 in 70:30 (vol:vol) DMSO:acetic acid), and the reaction was allowed to proceed for 2 h at 65°C. After the reaction, the excess labeling reagent was removed by an S-cartridge (QA-Bio). To analyze the 2AB labeled glycans, the mixture was separated on a C18 reversed-phase column (3 μm, 4 × 250 mm, Thermo Scientific, Waltham, MA, USA). The mobile phase was a mixture of 0.1% acetic acid in water (A) and 0.1% acetic acid in 10:90 acetonitrile:water (B). The gradient was from 30% B to 75% B in 135 min. The flow rate was 0.2 mL/min. The eluate was monitored on a fluorescence detector with excitation at 330 nm, and emission at 420 nm. RP-HPLC was performed using an Agilent (Santa Clara, CA, USA) Series 1100 binary pump system directly coupled to the mass spectrometer. During method development, in line mass spectrometer was used to confirm identity of glycans (33–35).

### Instruments

Experiments described in this manuscript were performed using Agilent 1100, Agilent 1290 and Waters HPLC systems equipped with diod array detector (DAD) or Variable Wavelength Detector (VWD). Capillary Electrophoresis (CE) experiments were performed on a Beckman PA 800 instrument.

Mass spectrometry experiments were conducted using a linear ion trap mass spectrometer (LTQ, Thermo Electron), equipped with an ESI source coupled directly to the HPLC instrument (33–35). All experiments were performed in the positive ion mode. Typically, LC/MS and tandem MS experiments were conducted using a spray voltage of 3.5 kV and a capillary temperature of 250°C. For the generation of tandem mass spectra, the parent ions were selected with an m/z window of 10, and the relative collision energy was set to 35%.

All data were acquired on qualified instrumentation operated by trained analysts.

## RESULTS

Development of the UBCI model involved three major steps: (1) an examination of historical in-house data from several qualification studies performed for different types of chromatographic and electrophoretic separations; (2) a series of repeatability tests signifying intrinsic variability of measured method precision and (3) a thorough evaluation of the signal and noise in chromatographic systems. Following the development of the model as it relates to method precision, which is a central feature of the model, we extended the model to all the performance characteristics of the method. Finally, we applied the model to a single chromatogram to assess the uncertainty of results, and compared it with historical results of qualification as a test of the validity of the model.

### Survey of Qualification Studies

Over the last several years, we have collected method performance characteristic data, as prescribed by the ICH Q2R1(6) guideline, for approximately 90 methods applied to as many as 20 different protein pharmaceuticals. Multiple sample types were often investigated for the same product. Examples of chromatograms and an electrophoregram for SE-HPLC, CEx-HPLC and CE-SDS methods, respectively, are shown in Supplementary Material Figure S1.

We retrospectively evaluated the data set and found that the values of many performance characteristics fall into very narrow ranges. An example of the statistical evaluation for these performance characteristics is presented in Table S1, Because data are not normally distributed, we have shown mean, median, range and 90 percentile. For certain sample types, we have performed an abbreviated method qualification (for which the evaluation of all performance characteristics/parameters was not evident or required) that resulted in an apparent incomplete data set, and this is reflected in a different “n” values for different performance characteristics.

**Fig. 1:**
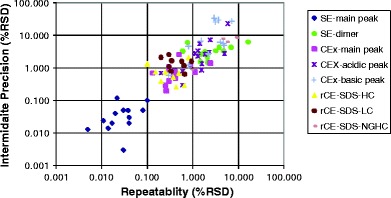
Relationship between repeatability and intermediate precision for three purity methods (SE-HPLC, CEx-HPLC, and rCE-SDS). Each point of the graph represents different protein analyte. Data were obtained in qualification experiments.

The repeatability and intermediate precision data for the SE-HPLC, CEx-HPLC, and CE-SDS methods is shown graphically in Fig. 1. Repeatability refers to the ability of the method to generate identical results for the same sample within a short time interval. Intermediate precision refers to the ability to replicate measurements by someone else, in a different laboratory, and/or different time interval. The graph indicates a linear relationship between repeatability and intermediate precision expressed in the form of RSD, with a slope approaching unity. However, for a given value of repeatability, the spread for intermediate precision is approaching one order of magnitude (note the log-log scale of the graph).Three distinct clusters are evident. The bottom-left cluster is represented by the precision of the main peak from SE methods. The middle cluster corresponds to precision of the main peak from ion-exchange and CE methods. The upper-right cluster represents the precision for minor peaks from all three methods. The level of the minor peaks ranges from a fraction of a percent in SE-HPLC methods to approximately 10% in CEx-HPLC. This close correlation between repeatability and intermediate precision could be a result of standardization of equipment to one or two brands to reduce the complexity of method transfer. This shows that performing both repeatability and intermediate precision studies may have limited value in exploring method performance.

The data shown in Fig. 1 corroborates the Horwitz relationship between the concentration of analyte and the precision of the method expressed in RSD (36,37). Minor peaks, for which concentration is one or two orders of magnitude lower than the main peak, showed significantly higher RSD than the main peak. However, the observed relationship may not strictly follow the canonical rule that a change in concentration of two orders of magnitude corresponds to a two-fold change in precision (38).

### Repeatability

We designed experiments to evaluate the consistency in the determination of method precision (repeatability) for the same sample. The sample was analyzed using the same instrument, by the same analyst, on two different days. To maximize information obtained from a limited number of experiments, we evaluated the output of two different methods with well resolved multiple peaks: peptide and glycan maps. Large amounts of peptide digest and 2AB derivatized glycans were prepared. The same sample was used in all experiments described below. The peptide maps show 9 peaks, and were monitored using a UV detector at 215 nm. Additionally, the chromatograms for peptide maps were acquired at different acquisition rates to introduce variability in the form of a different level of observable noise. Glycan maps were used to monitor 16 well resolved glycan forms, and were monitored using a fluorescence detector signal. Figure S2 shows examples of the peptide and glycan maps. Table S2 summaries the overall design of the experiment.

The experiment was designed to test the hypothesis that there should be no significant difference between the method repeatability of two sets of measurements generated on two different days. We used a statistical F-test to evaluate this hypothesis (15). For this hypothesis to be true, the ratio of the two sample variances should be less than the critical F-values defined by two values of degrees of freedom. The variance of purity measurements for individual peaks (analytes) of the peptide map was calculated from analysis of triplicate experimental runs on both days. For the peptide map, the degrees of freedom for both the numerator and the denominator of 2 for the F-test resulted in an F-critical = 19.0 at a 95% confidence level. For glycan maps, the variance for individual peaks was calculated from 3 replicates on day 1 and 9 replicates on day 2, corresponding to degrees of freedom of 2 and 8, respectively. In this situation, F-critical will assume values of 4.5 and 19.4, respectively, depending on which value of variance was used as the denominator. The results of these experiments and the value of the calculated F-critical are shown in Table I.

**Table I:** Results of Experiments Comparing Method Repeatability Using an F Test. (Red Font Indicates Non-Passing Values)

A-Peptide map (3 replicates, 2 degrees of freedom for both the numerator and denominator of the F-test, F_crit_ = 19.0)									
	1 Hz			5 Hz			20 Hz		
	Variance		F_ratio_	Variance		F_ratio_	Variance		F_ratio_
Peptide#	Day 1	Day2		Day 1	Day 2		Day 1	Day 2	
1	5.78E-07	8.19E-07	1.42	2.25E-07	6.31E-07	2.81	5.83E-07	3.07E-08	18.95
2	1.67E-08	5.65E-09	2.96	1.36E-08	6.42E-08	4.72	2.52E-08	1.60E-07	6.36
3	1.09E-08	1.22E-09	8.98	4.66E-10	2.49E-08	53.50	1.08E-09	5.49E-08	50.76
4	2.53E-09	5.33E-09	2.10	4.01E-09	1.84E-08	4.59	3.15E-09	2.32E-08	7.37
5	8.12E-08	1.45E-07	1.78	3.76E-08	3.55E-08	1.06	3.60E-09	2.26E-08	6.27
6	2.34E-08	2.76E-08	1.18	1.29E-08	3.46E-08	2.69	6.22E-10	5.00E-08	80.36
7	6.66E-08	1.95E-08	3.42	1.19E-09	6.29E-08	53.02	1.61E-08	1.13E-07	7.03
8	8.60E-09	3.77E-08	4.38	2.59E-09	9.90E-09	3.82	9.94E-09	1.55E-08	1.56
9	4.29E-08	7.36E-08	1.72	2.75E-07	3.19E-07	1.16	7.07E-07	2.18E-07	3.24
B, Glycan map ( 3 or 9 replicates, correspond to 2 and 8 degree of freedom respectively)									
Glycan #	3 repl./Day1	9 repl. Day2	F _ratio_	F _crit_					
1	6.80E-10	1.61E-09	2.37	19.4					
2	7.66E-10	7.32E-09	9.56	19.4					
3	4.08E-10	2.50E-10	1.63	4.5					
4	1.76E-09	1.18E-09	1.49	4.5					
5	7.89E-09	4.19E-09	1.88	4.5					
6	3.71E-09	4.53E-08	12.21	19.4					
7	1.60E-08	1.40E-07	8.75	19.4					
8	2.31E-08	5.54E-08	2.40	19.4					
9	1.39E-07	1.47E-07	1.06	19.4					
10	1.82E-10	2.67E-09	14.68	19.4					
11	1.02E-08	1.17E-08	1.15	19.4					
12	1.20E-08	9.81E-08	8.16	19.4					
13	8.44E-09	1.77E-08	2.09	19.4					
14	1.65E-08	2.04E-07	12.38	19.4					
15	7.09E-08	5.28E-08	1.34	4.5					
16	6.56E-08	3.63E-06	55.33	19.4					

The results show that precision measurements for certain pairs of experiments were significantly different, and in 12% of cases (4 out of 27 for the peptide map and 1 out 16 for the glycan map) the ratio of variances exceeded the value of the corresponding F-critical at the 95% confidence level. It can be concluded that in certain situations a significant difference does exist between precision of purity for certain peaks (analytes) measured by the same method on two different days. 60% of F test results had the ratio of two variances greater than 3 in Group A and 36% of them had that ratio in Group B. 19% of the experiment results had the ratio of two variances greater than 10 in Group A and 25% of them in Group B (21% overall). The F test was conducted based on limited sample sizes, and may have an imperfect statistical power. However, this is a practically significant indication that the assay variability for the same sample is not constant over multiple days, even when the assay is performed on the same instrument and by the same analyst. It appears that a significant variability of precision is an intrinsic property of chromatographic methods.

### Examination of Noise in Chromatographic Systems

Stimulated by the work of Hayashi and Matsuda on FUMI theory (22,25), which relates the stochastic properties of background noise to method precision, we turned our attention to chromatographic noise (39,40) with the intention of finding an explanation of these intriguing observations regarding precision, specifically, a significant variability in the determination of precision for individual analytes, and a close relationship between repeatability and intermediate precision. The FUMI theory requires parameterization of the Fourier transformed noise (power density spectrum) to compute the value of the noise parameters for the chromatographic measurements (22,25). However, the power density spectrum showed distinct regular features that made the spectrum difficult to parameterize per the Hayashi and Matsuda method. The complexity of the power spectrum can be attributed to the proprietary manipulation of the signal by hardware and software manufacturers.

**Fig. 2:**
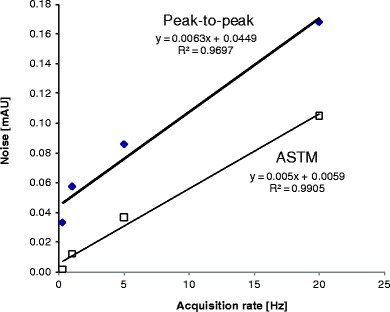
Relationship between the level of noise (solid diamonds represent peak-to-peak noise, open squares represent ASTM noise) and data acquisition rate.

Many commercial chromatographic software packages can extract and report information about the noise in different ways. The most popular are peak-to-peak noise and ASTM noise (which is based on ASTM standard E 685–93) (41). Peak-to-peak noise is a transient measure of the amplitude of the noise, while the ASTM standard provides an averaged value of the noise. Figure 2 shows the change of the amplitude of peak-to-peak and ASTM noise as a function of the acquisition rate, where the data was extracted uniformly from chromatograms of the peptide maps. The graph illustrates a linear increase of the observed noise with an increase of the acquisition rates in the range of 0.25 to 20 Hz. Parallel regression lines indicate a linear relationship between peak-to-peak noise and ASTM noise. A similar general relationship has been observed for more than 200 noise measurements extracted from a variety of chromatograms (data not shown). It appears that the reported peak-to-peak noise is roughly 1.3-1.5 times larger than the ASTM noise, which is consistent with the definition of ASTM noise providing an “averaged” value of the noise instead of the “local” peak-to-peak noise that can be transiently overestimated.

### Signal to Noise Ratio and Precision

**Fig. 3:**
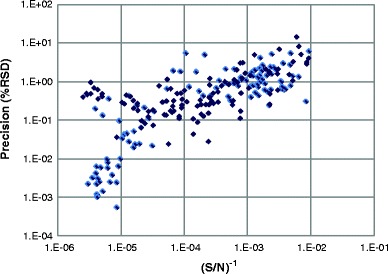
Relationship between precision and S/N ratio for chromatographic purity methods.

Inspired by Dolan’s rule of thumb (42), $RSD \approx \frac{{50}}{{S/N}}$, we have explored the connection between method precision and the signal to noise (S/N ) ratio. Figure 3 shows the experimental affiliation (in log-log scale) between RSD of the methods and (S/N)^−1^ for more than 200 chromatographic peaks (from SE-HPLC, CEx-HPLC, peptide maps, and glycan maps). The graph shows that the decrease in S/N ratio results in the lower precision of the method (higher RSD). For the same S/N ratio, the spread in the precision approaches 3 orders of magnitude, indicating that factors other than S/N ratio significantly influence the precision of purity measurements.

### Assessment of Precision for Purity Methods

Previous experiments indicated that the S/N ratio is not a single factor influencing the precision of purity measurements, which steered us toward the development of a more comprehensive model of precision for purity measurements that takes into account multiple sources of potential error as well as other factors, described below.

Let us consider a chromatogram with two or more peaks, as represented in Figure S3a.

The purity, relative abundance, (P_1_) of the first analyte (component 1) will be expressed as a function of two independent variables R_1_ and A_1_ as follows:

4 $$ {P_1} = \frac{{{A_1}}}{{{R_1} + {A_1}}} $$

where A_1_, represents the area of the first component, and R1 represents the area under all other peaks on the chromatogram (R_1_ = A_2_ + A_3_ in Fig. S3). Relative abundance of subsequent components can be expressed in an analogous way. The variance of P_1_ can be approximated by the following equation (detailed proof can be found in the Supplementary Material).

5 $$ V({P_1}) \approx \frac{{E{{({A_1})}^2}V({R_1}) + E{{({R_1})}^2}V({A_1}) + 3V({A_1})V({R_1})}}{{E{{({A_1} + {R_1})}^4}}} $$

where E(A_1_) is the expected value for the area under peak 1 (A_1_), E(R_1_) is the expected value for the area of all peaks except the peak of interest, A_1_, E(A_1_ + R_1_) is the expected value for the total area expressed as A_1_ + R_1_, V(A_1_) is the variance of A_1_, and V(R_1_) is the variance of R_1_.

Expected value can be derived through statistical analysis of measured area under the peak and can be approximated by averaging values of this random variable. The value accounts for errors associated with the measurement.

If the A_1_ area is very small (A1<<R1), typically order of magnitude smaller than R_1_ (equivalent to A_1_ less than 10% of the total area), the above equation can be simplified.

6 $$ V({P_1}) \approx \frac{{V({A_1})}}{{E{{({R_1})}^2}}} $$

Equations 5 and 6 show that the variance of purity depends on variances and expectation values for the peak area of analytes, where both statistics behave in an uncorrelated manner. This indicates that the *precision* of purity will be influenced by the absolute amount of the injected analyte.

The expected values of A_1,_ R_1_, and A_1_ + R_1_ can be approximated by computing averaged values. Therefore, it is critical to assess variances for A_1_ and R_1_. In our model, all peaks are well resolved, and can be treated as independent variables. Therefore, the variance of R_1_, can be expressed as the sum of variance of individual peaks.

7 $$ V({R_1}) = V({A_2}) + V({A_3}) + \ldots $$

The true area under each individual peak can be expressed as the area of the theoretical peak and a series of independent errors impacting the measurements. We are assuming that samples are analyzed without the introduction of preparation errors (e.g., dilution error)

8 $$ {A_1} = A_1^0 + {E_{{Inj.}}} + {E_{{Integration}}} + {E_{{Noise}}} $$

where $A_1^0$ is the theoretical area under the peak (constant for the given sample, and does not account for any errors), *E*_*Inj.*_ is the error of injection influencing the peak area, *E*_*Integration*_ is the error of numerical peak integration, and *E*_*Noise*_ is the error of peak integration influenced by baseline noise.

Therefore, assuming that all the components are independent

9 $$ V({A_1}) = V(Inj.) + V(Integration) + V(Noise) $$

Injection error (E_Inj._) is typically expressed in the form of RSD by the instrument makers, and is generally listed in the instrument specification table. Therefore, the variance of injection can be expressed by the following equation

10 $$ V(Inj.) = {(A_1^0)^2} \times {(RS{D_{{Instr.}}})^2} $$

where *RSD*_*Instr*._ = Instrument precision (injection precision) taken from the instrument specifications.

The error of peak integration is related to the numerical integration of the area under the peak, and constitutes a broad family of algorithms for calculating the numerical value of a definite integral. A large class of integration algorithms can be derived by constructing interpolating functions. The simplest method of this type is to let the interpolating function be a constant function within the integration subinterval, and is called the *midpoint rule* or *rectangle rule* (43). Assuming that the shape of the chromatographic peaks is described by a Gaussian function, the error of integration, using the rectangular rules (Fig.S3b), can be expressed by the following equation (43).

11 $$ V(Integration) = V\left[ {\frac{{{w^2}}}{{2n}}\max f\prime (t)} \right] = \frac{{{{\left[ {\max f\prime (t)} \right]}^2}}}{{4{\nu}{^2}}}V(w) $$

where *w* is the width of the integration domain (peak width at the base in sec), V(w) is the variance of the integration domain, which must be known or experimentally determined, n is the number of points across the integration domain of peak 1 (Fig. S3a), which can be expressed as n = *w ν*, *ν* is the frequency of acquisition (points /sec), t is time, and *max f’(t)* is the maximal value of the first derivative of the function describing the peak shape. We assume that peaks are described by Gaussian functions, for which *maximal value of the first derivative* is at the deflection point one σ from the apex; therefore

12 $$ \max f\prime (t) = \frac{{A_1^0{e^{{ - 1/2}}}}}{{\pi {\sigma^2}}} $$

where $A_1^0$ was described earlier as theoretical area under peak 1 (constant for the given sample) and σ is the dispersion (standard deviation) of the Gaussian distribution expressed in the units of time domain.

Therefore, the variance of the numeric integration can be expressed as:

13 $$ V(Integration) = {\left[ {\frac{{A_1^0{e^{{ - 1/2}}}}}{{2\nu \pi {\sigma^2}}}} \right]^2} \times V(w) $$

Different instrument makers may use different algorithms, but application of the rectangular rules most likely will approximate the upper bound of the numerical integration variance.

The third component contributing to the error in determination of the peak area is the noise of the system. Noise is a low-level high frequency signal, and has the property that when it is integrated over a longer period of time, it averages to zero. Therefore, assuming that the baseline of integration is accurately determined, the contribution of the noise to peak area can be considered negligible. However, noise can interfere with accurate determination of the baseline, which can lead to a significant variability in the measured peak area, as illustrated in Figure S3c. The same peak of the analyte can be integrated differently, depending on the “position” of the noise *vs.* the signal. Introduced integration bias can be defined as extra area added or subtracted to the theoretical area of the peak, depending on the (arbitrary) established baseline. The maximum area added or subtracted is a rectangle that can be defined as w × N, where N represents the amplitude of the noise, and w represents the width of the integration domain. Based on hypothesis that the major contribution to noise is given by the photon counting in the detector (44), we assume a Poisson distribution of the noise, which has a unique property that the numerical value of variance is equal to the expected value.

Therefore,

14 $$ \matrix{ {V(Noise) = V(wN) = E{{(w)}^2}V(N) + E{{(N)}^2}V(w) + V(w)V(N) = } \hfill \\ { = E{{(w)}^2}E(N) + E{{(N)}^2}V(w) + V(w)E(N)} \hfill \\ } $$

Typically, V(w) is significantly smaller than the expected value E(w); therefore, the second and third term in the above equation will be much smaller than the first term, and can be omitted, as follows:

15 $$ V(Noise) \approx E{(w)^2}E(N) $$

Combining together [10], [13], and [15], the maximal value of variance of each individual peak can be expressed by the following equation

16 $$ V({A_1}) \approx {\left( {A_1^0RSD} \right)^2} + {\left( {\frac{{A_1^0{e^{{ - 1/2}}}}}{{2\nu \pi {\sigma^2}}}} \right)^2}V(w) + E{(w)^2}E(N) $$

We do not know the exact value of $A_1^0$, but it can be approximated with the averaged value of A_1_. Similarly, expected value of noise, *E(N)* ,can be approximated with the averaged value of ASTM noise. The value of dispersion of chromatographic Gaussian peaks (σ) can be approximated from the average of peak width at the half height by using the following relationship: σ = w/2.355 (45). Now, to compute the variance of the purity determination, we will need to return to equations 5 and 7, and substitute in their values obtained from equation 16. In the special cases of small minor peaks (<10%), equations 6 and 7 can be used. Virtually all of the information that is needed to calculate the precision of purity measurements can be extracted from the current chromatogram; therefore, we call the developed concept a model of Uncertainty Based on Current Information (UBCI).

We applied the UBCI model to calculate the maximum imprecision of purity measurements for individual peaks in our methods: glycan and peptide maps. Glycan and peptide maps were chosen because they show well resolved peaks with wide ranges of intensity and peak width, which allows for quick evaluation of the model. In the case of peptide and glycan maps the term purity refers to relative abundance of the particular peptide or glycan. As stated previously, peptide maps were monitored using a UV detector, while for glycan maps, we used a fluorescence signal. To introduce additional variability in the form of a different level of observable noise, we collected chromatograms at different acquisition rates. Table S3a summarizes the design of the experiment.

All measurements were performed in replicates, creating an opportunity to calculate the precision using traditional statistical methodology, and the prediction of maximal uncertainty using the UBCI model.

First we calculated the variance of area under each individual peak using equation 16, which requires knowledge of the following variables: peak area, peak width at the base (width of integration domain), peak width at the half height, the level of baseline noise, the acquisition rate, and the precision of injection for the HPLC instrument.

**Fig. 4:**
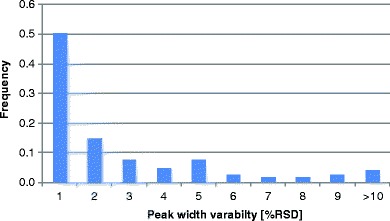
Distribution of the variance of the integration domain. Experiments outlined in Table S3 were used in the analysis. Total of 206 analytes were evaluated.

Replicate analyses were used to calculate the variance of the integration domain (peak width at the base). The variability of the integration domain expressed in the form of RSD showed a characteristic distribution (Fig. 4). Data indicates that in 50% of the scenarios the RSD of the integration domain is less than 1%; in 75% of the scenarios, RSD of the integration domain is within 3%.

The expected values of all variables were approximated from averages. We used ASTM noise provided by the Agilent ChemStation software to approximate the amplitude of the noise. The acquisition rate was extracted from software settings embedded into the data files. The injection volumes exceeded 5 μl in all experiments; therefore, the imprecision of such injections should not exceed 0.5% RSD, based on the HPLC specifications (46). In the next step we used equation 7 to calculate the variance of complementing peaks (sum of all peaks except peak of interest) on the chromatogram, and finally we used equation 5 to calculate the variance of the purity measurements for each individual peak.

**Fig. 5:**
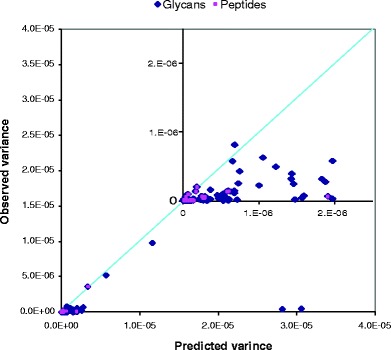
UBCI Predicted *vs.* observed variance of purity for individual peaks on peptide and glycan maps. Solid line represents equivalency between these two values.

A total of 104 calculations and predications were conducted. The observed variance for these measurements *vs.* predicted uncertainty in the form of variance is shown in Fig. 5. The results show that in all but a few cases, the observed variance did not exceed the predicted maximal variance, which is visualized by the fact that virtually all points fall below the line with a slope equal to one. The two points farthest away from the origin (Fig. 5) are associated with the variance of peak 9 in the peptide map. The peak was integrated very broadly (peak width at the base exceeded 20 times peak dispersion), and in one case, (farthest point) variability of the integration domain was approaching 40%. These results indicate that the uncertainty of purity measurements can be controlled by integrating in a consistent manner and minimizing the width of the integration domain (47,48).

In addition to the model system of peptide and glycan maps, we applied the UBCI model to size exclusion and ion exchange chromatographic separations. We applied this analysis to the well resolved peaks. In the case of CEx-HPLC, as exemplified in Figure S1, we omitted computing precision for the basic peaks that are small and poorly resolved. Six different protein products were evaluated, including mAbs and fusion proteins. A total of 101 predictions of precision were computed. The design of these experiments is summarized in Table S3b. When plotted as observed *vs.* predicted variances, the graph looks similar to the results presented in Fig. 5 (data not shown), which demonstrates the general utility of the UBCI model.

**Fig. 6:**
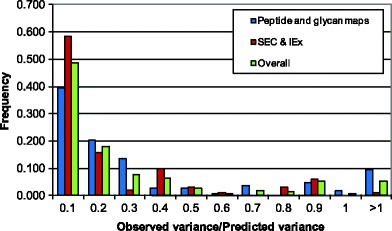
Distribution of the observed *vs.* UBCI calculated variance of purity measurements for different types of methods.

An alternative representation of all results (SE-HPLC, CEx-HPLC, peptide and glycan maps) is presented in the form of the distribution of the ratio of the predicted variance *vs.* measured (observed) variance. A total of 205 points were analyzed. The ratio represents the measurement of the agreement between the predicted and measured uncertainty. Figure 6 shows the frequency of the ratio for peptide and glycan maps, SE-HPLC and CEx-HPLC, and overall distributions, respectively. All three distributions look very similar. The result shows that frequently (approximately in 50% of cases), the observed variance of purity measurements is less that 1/10 of the predicted variance. Larger ratios are less frequent, creating a characteristic stochastic distribution. This is related to the fact that a small number of replicates (typically triplicates) are used to calculate sample variance, which on some occasions may be significantly different from the population variance (15).

The results shows that the UBCI model can successfully predict the precision (in the form of variance) of the results based on the information imbedded in chromatograms. The estimate of precision corresponds to the uncertainty under the most unfavorable conditions, including the highest variability of injection, maximal numeric integration error, most plausible variability of the peak width, and most unfavorable contribution of the noise to the error of peak integration. The model offers a dynamic live assessment of precision under the current conditions (consumables, hardware configuration, and software settings) of the method.

### Specificity

After successfully addressing method precision dynamically, we turned our attention to the six remaining performance characteristics (specificity, linearity, range, accuracy, DL and QL) to determine whether they can be addressed based on the current chromatographic information.

**Fig. 7:**
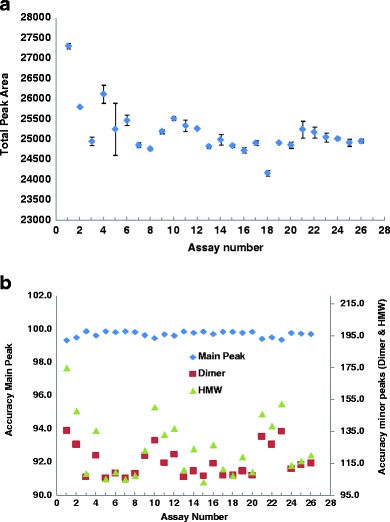
Example of trending protein recovery for SE-HPLC method as an expression of (**a**) specificity, and (**b**) accuracy.

The specificity of analytical methods is typically assessed by examining system interference with the detection and quantification of analytes. Part of this evaluation is the determination of protein recovery using equation 1 (see Materials and Methods) (29,30). With such an approach, the specificity of the method can be assessed real-time in every assay, and reflects dynamically the change in status of consumables (columns and mobile phases) and hardware. The use of the equation requires the knowledge of the extinction coefficient for the protein, which can be calculated from its amino acid composition (31) or determined experimentally. The extinction coefficient can be considered constant for a given protein. Therefore, fluctuation of the area under the peak from the system suitability reference material can be a good reflection of protein recovery. Figure 7a shows an example of trending for the total area using a chromatogram for the SE-HPLC method.

It should be noted that the extinction coefficient of a protein may change as the function of pH (29,49). Therefore, direct comparison of the recovery in the neutral pH size exclusion method with the recovery in an acidic reversed-phase separation may not be valid due to differences in the operating pHs of the methods. The difference may not necessarily reflect the discrepancy in recovery, but rather shows pH dependent changes of spectroscopic properties of the protein.

### Accuracy Assessment of Purity Methods for Biopharmaceuticals

The determination of accuracy for protein purity methods presents significant challenges. Since it is difficult to establish orthogonal methods for proteins to measure the same quality attribute, it is hard to assess the truthfulness of the accuracy measurements. For example, although SE-HPLC results can be verified by analytical ultra centrifugation (AUC) techniques these techniques are based on very different first principles, and may not provide comparable results (50,51). Therefore, in most cases, the accuracy of purity methods for proteins is inferred when other performance characteristics meet expectations, which is consistent with the principles of ICH Q2R1 (6).

Alternatively, accuracy can be assessed by examining the results of purity calculations for the reference material used to test system suitability. By convention, the reference standard is well-characterized material, for which true values of quality attributes have been established through a very detailed characterization. Therefore, the ability to recover these true values during system suitability injections can be considered as a means for determination of the accuracy of the method. Figure 7b shows an example of trending accuracy for a SE method over several months. Such expression of accuracy allows for its live assessment.

17 $$ Accuray = \frac{{{P^{{SS}}}}}{{{P^{{RS}}}}}100\% $$

where P^SS^ is the % purity for individual analyte obtained during system suitability and P^RS^ is the % purity for individual analyte obtained during reference standard characterization.

### Linearity and Range of Major and Minor Species

**Fig. 8:**
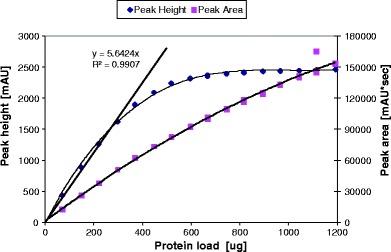
Load Linearity for SE-HPLC method designed for mAb.

Linearity and range are typically assessed in a complex experiment demonstrating a linear change of peak area with analyte concentration. Such experiments can be considered as re-examinations of the Beer-Lambert law for the particular hardware configuration. Different amounts of mAb were analyzed by the SE method ranging from 70 μg to 1200 μg. The separation was monitored by a UV detector at 280 nm, with the linear absorbance range of 2 AU (per detector specifications (52)). Peak height and peak area were plotted against the total amount of protein loaded. The resulting graph is show in Fig. 8. As seen from the graph, the method comes close to the upper limit of the linear range at the protein load of approximately 300–400 μg as monitored by the peak height. At that load, the height for the main peak approaches the upper specification limit of the detector, and then levels off. However, the loss of linearity is not very apparent from the trace of peak area *vs.* load. This is due to increased peak width with larger amount of injection, causing column and detector saturation. This indicates that plotting peak height *vs.* load is enhancing the assessment of method linearity, and suggests that the method linearity could be inferred if the tallest analyte peak is within the working range of the currently used detector. The peak of the dimer (minor peak) showed linear behavior with respect to the height and area over the entire range of 70–1200 μg of total protein load (data not shown). A similar behavior was observed for other mAbs analyzed by different methods at different wavelengths (220 and 280 nm). In this context, the detector’s specification limit could be considered as the upper limit of the range, while detection limit (DL) expressed in mAU can be considered as the lower limit of the range of the method. Both of these characteristics, linearity and range, can be assessed real-time for every hardware configuration and light source.

#### Detection and Quantification Limits

Practical use of the Detection Limit (DL) is related to the decision whether to integrate or disregard the peak on the chromatogram, and is very closely related to the evaluation of the S/N ratio. ICH guideline Q2R1 defines the DL as the minimum level of analyte which can be readily detected (6). For chromatographic methods, a signal to noise ratio of 3 is generally considered acceptable for estimating the detection limit. This approach is straightforward and allows for dynamic assessment of DL for each individual chromatogram based on the extracted information about the amplitude level of the noise.

Assessment of quantification limit (QL) is required for most analytical methods developed to monitor minor species that are considered critical quality attributes for the product. ICH defines QL as the minimum level of analyte which can be quantified with acceptable accuracy and precision (6). Practical use of QL is related to the decision on whether to report the results of tests. Here we present a challenging example of QL determination for the acidic peak separated by a CEx-HPLC method.

Initially, we used the most commonly practiced statistical approach to estimate the QL for the acidic peak resolved by cation exchange chromatography (Figure S1c). This approach is associated with the use of well known equations (6,53):

18 $$ QL = 10 \times \frac{{SD}}{S} $$

where SD = standard deviation of response, S = slope of calibration curve (sensitivity).

The slope and SD were obtained from linear regression of the results from a blending experiment by plotting % of acidic peak *vs.* the recorded response as integrated area under the peak at 280 nm (Fig. S4). In all blending experiments, we preserved the total nominal load defined by the method (120 μg of total protein). The experiment was performed by mixing our reference standard containing approximately 5% of the acidic form with other material containing up to 50% of the acidic form.

The ICH guideline Q2R1 recommends using standard deviation of the regression line or y-intercept of the regression line. In this case, values of standard deviation for the regression line and y-intercepts were very different, and resulted in the computed QL of 4.7 and 2.8%, respectively (Table S4). Such large values seem to be “artificially” inflated (unrealistic), indicating that our method may not be able to quantify the level of acidic peak in the purified product. Therefore, we attempted to estimate the QL for the acidic peak based on S/N ratio, which previously showed a good agreement with canonical methods (32).

19 $$ QL = 10 \times \frac{N}{S} \times P $$

where *N* is the peak-to-peak noise, *S* is the acidic peak height, and *P* is the % purity of the peak.

Table S5 shows the results of three experiments performed at the nominal load for a product containing approximately 5% of the acidic form. The calculation based on S/N ratio yielded a QL of approximately 0.6%, which is almost one order of magnitude lower than the previous assessment. The new value of QL was consistent with the ICH designation of QL, which requires a S/N ratio of 10 in order to quantify the peak with confidence. The discrepancy in estimates could be related to the fact that the blending experiment was performed in the range significantly above the QL, and skewed the assessment of SD of the residual and y-intercept for the regression line. In many situations, it is extremely difficult (sometimes impossible) to obtain pure forms of protein variants, which limits the practical range of the blending experiments. Therefore, dynamic assessment of the QL based on the S/N ratio (32) offers a quick and practical assessment of the QL for purity methods.

Using our UBCI model, we estimated the maximal imprecision of the measurement, assuming that the acidic peak was at the QL level, 0.6%. Also, we assumed that all parameters are the same as in the previous experiment (used to compute dynamic QL). The predicted variance of purity measurement was 0.0000156, which translates to 7.8% RSD. This is close to the anticipated 10% RSD for analytes at the QL level, assuming concentration homoscedasticity (23). The homoscedasticity means that the measurement of variance is invariant of analyte concentration. Our model does not require such assumption and may provide more realistic assessment of maximal imprecision near the QL.

### Practical Application of the UBCI Model

We have applied all aspects of the UBCI model (current chromatographic information) to assess uncertainty of the generated results obtained by a single analytical test (expected values in all equations were substituted with the results of a single experiment) We compared these results with those from historical qualification. The historical qualification was based on ICH Q2R1, and required approximately 101 independent experiments/injections, while dynamic assessment of performance is based on information extracted from a single chromatogram and the system suitability data accompanying the analysis. Figure S1a shows a SE chromatogram used to extract all the necessary information, which includes peak area, peak height, peak width at half height and at the base, ASTM noise, acquisition rate, and injection volume. 4 μl injection volumes were used to derive a 1% RSD of injection variability from the instrument specifications (46). To assess the specificity, the chromatogram was compared to the blank chromatogram. Table II lists all these parameters and the resulting output of the expanded UBCI model. We compared the values of performance characteristics estimated from a single chromatogram with historical qualification data for the method in Table III. The comparison shows that the UBCI model provides equivalent information about method performance.

**Table II:** Parameters Extracted from Single Chromatogram

	Parameter of the peak	HMW (pre-peak 1)	Dimer ( pre-peak 2)	Main
Input	Area [mAU*s]	28.118	384.102	22703.904
	Height [mAU]	0.801	10.185	871.261
	Wight at half height [min]	0.543	0.550	0.380
	Width at the base [min]	1.119	1.853	5.627
	Variability of peak width at the base [%]	RSD = 3% ( see Fig. 4)		
	ASTM noise [mAU]	0.00482		
	Acquisition rate [Hz]	2.5		
	Instrument Injection RSD [%]	1% for less than 5 μl (4 μl was injected)- see text		
Output	Purity	0.0012 (0.12%)	0.0166 (1.62%)	0.9822(98.22%)
	Variance of area (eq.16)	21.7388	63.2849764	14434
	Variance of purity (eq. 5)	4.06E-08	1.22E-07	1.62E-07
	DL [mAU]	0.01446	0.01446	N/A
	QL [%] (eq.19)	0.00007 (0.007%)	0.00008 (0.008%)	N/A

**Table III:** Comparison of the UBCI Prediction and Historical Qualification Results

Performance characteristic	Analyte	Historical Qualification	Live Validation	Comments
Specificity	all	No interference Recovery = 97.2%	No interference Recovery = 91.1%	Equivalent
Repeatability RSD	Main	0.02%	0.04%	Prediction and historical data are consistent
	Dimer	1.55%	2.10%	
	HMW	5.60%	16.57%	
Linearity	all	R2 = 0.9999	Passed (main peak =871.261 mAU, lower than specifications)	Historical data and current information expressed by different parameters. Tallest peak within detector’s linear range
Range	all	Tested for 50–150% of the nominal load	0.01446-2000 mAU	Assuming first principles of the Beer- Lambert law, the methods is linear within DL and detector’s upper range
Accuracy	Main	100.0%	99.9%	These measures are not equivalent (see text)
	Dimer	100.1%	106.7%	
	HMW	100.2%	101.7%	
DL	Dimer	0.03% (static)	0.01446 mAU	Historical and prediction data expressed in different units. Dynamic DL linked to the level of noise
	HMW	ND	0.01446 mAU	
QL	Dimer	0.1% (static)	0.008%	Dynamic QL reflects the current status of the instrument and consumables, and is linked to the level of noise. Such value can be significantly different form historical data
	HMW	ND	0.007%	

## DISCUSSION

The ICH Q2 guideline requires that an analytical method be validated for commercial pharmaceutical and bio-pharmaceutical applications to demonstrate the method’s suitability for product disposition and stability testing (if applicable). Frequently, validation is done only once in the method’s lifetime. This is particularly of concern when the future testing is performed on an instrument with different technical characteristics, in different geographic locations, using different consumables, different analysts, etc. This concern is exacerbated by the requirement for modern pharmaceutical and bio-pharmaceutical companies to seek regulatory approval in multiple jurisdictions, where the instrumentation, consumables, and scientific staff experience may be very different than in the place where the drug was developed. These considerations raise questions about the value of the current format of the validation studies conducted by the industry. Moreover, it is not clear how the validation data obtained using existing methodologies should or even could be used toward the assessment of uncertainty of the future results.

To overcome this limitation, we have developed the comprehensive UBCI model. We have shown that UBCI can be successfully applied to assess all performance characteristics of purity methods. This assessment can be performed live based on the current information embedded in the chromatogram or electrophoregram of the tested sample and from the accompanying system suitability data. In Table III we compare the results of the live assessment of uncertainty with historical qualification results, which were generated on an equivalent instrument approximately a year prior to performing the test analysis. We showed that the performance assessment obtained using UBCI correlates well with the historical data acquired on one brand of instrumentation. The historical value of RSD for high molecular weight (HMW) specie (5.60%) is notably lower than the predicted precision (16.57%). This is related to the fact that UBCI model predicts the upper bound of precision, which could make the difference more apparent for small and broad peaks. Also, linearity, range, and accuracy were expressed differently than for the historical data.

This live assessment approximates the maximal uncertainty of the measurement associated with the actual conditions of analysis (test). The obtained precision corresponds to the uncertainty under the most unfavorable conditions, including the highest variability of injection, maximal numeric integration error, expected variability of the peak width, and the most unfavorable contribution of the noise. UBCI shows that the uncertainty of results is not only a function of the method (composition of the mobile phase, gradient, flow rate, temperature), but also is influenced by the hardware associated with the execution of the method (pump pulsation, detector range, status of the lamp, etc.), and the software settings used to acquire the output in the form of chromatograms. Information about these parameters can be extracted from individual chromatograms; therefore, the assessment of method performance characteristics (uncertainty) can be performed real-time, which can be considered as a ‘live validation’ associated with each individual test result. We demonstrated that the model is suitable for well-resolved peaks, and will require further refinement for poorly resolved, overlapping peaks (with resolution <1). We intend to continue our effort in this direction. Such refined model may have practical utility in improving the assessment of uncertainty of results generated by ion exchange methods, which typically display poorly resolved peaks.

The most essential aspect of assessing the uncertainty of purity measurements is the precision. In our model, the variance of purity measurements is a function of the reproducibility of the injector, numerical integration, and baseline noise, which is reflected in three terms, as shown in equation 16. Analysis of the equation offers multiple ways to minimize the variance of purity measurements. The first term of the equation can be minimized by selecting a HPLC instrument with low imprecision of injection. The second term of the equation can be zeroed if the variance of the integration domain is equal to zero, or can be minimized with the use of consistent integration practices. Additional factors minimizing the influence of the second term include a narrow peak width at half height (sharper peaks), and an optimal acquisition rate. A faster acquisition rate will minimize the contribution of this term but it will intensify the noise, which will increase the value of the next term. The influence of the third term can be minimized by decreasing the baseline noise and the width of the integration domain (48,54), which is an expression of the S/N ratio for Gaussian-shaped peaks. Overall, high fidelity HPLC, consistent integration practices, and a high S/N ratio can significantly increase the precision of purity measurements.

The UBCI model shows that the most probable variance of experiments is 1/10 of the variance predicted by the UBCI model, even when the measurements are conducted on the same instrument by the same analyst. This has been corroborated by the F-test experiment, which shows that in 21% of cases, the repeat of the experiment executed under the same conditions can differ by an order of magnitude or more. Therefore, one should use extreme caution in using precision as an acceptance criterion in studies involving method equivalency, technology transfer, etc. Additionally, this suggests that the value of precision obtained during method qualification or validation should be treated with a healthy dose of “skepticism” when assessing future uncertainty.

Laboratories that work in a GMP environment are required to produce extensive documentation to show that the method is suitable. Pharmaceutical and bio-pharmaceutical companies “religiously” adhere to these requirements, which inundates industry with an avalanche of validation work that has questionable value toward the future assessment of uncertainty. Our UBCI model of uncertainty provides an alternative that has the potential to reduce the work required to demonstrate method suitability and, in turn, provide greater assurance of the validity of the results from the specific analysis in real time.

Recently, the industry has begun adopting the concept of science based Quality by Design (QbD) to several aspects of the pharmaceutical lifecycle (55). The basic concepts are described in ICH guidelines Q8, Q9 and Q10 (56–58). Our methodology is consistent with the spirit of QbD. Perhaps the time is right for the industry to consider the use of a combination of sound science and reasonable risk assessment to change the current practice of the retrospective use of method validation to the new paradigm of live validation of purity methods based on the current information embedded in the chromatogram. While the proposed model may be contentious, as it challenges the way in which method qualification and validation have been traditionally performed, the model is worthy of consideration because it addresses some of the existing limitations of the current method qualification/validation paradigm.

## Electronic supplementary material

Below is the link to the electronic supplementary material.

ESM 1 (DOC 1.05 MB)
