# Supplementary material for: Uncertainty Estimates of Purity Measurements Based on Current Information: Toward a “Live Validation” of Purity Methods
Source: Pharm Res. 2012 Aug 15;29(12):3404–19. doi: 10.1007/s11095-012-0836-z (PMC3497960; doi:10.1007/s11095-012-0836-z)
Supplement: Supplementary file 1 — (DOC 1.05 MB) [file 11095_2012_836_MOESM1_ESM.doc]

## SOME GENERAL CONSIDERATIONS

Let *N* be noise such that **E**[*N*]=0 and variance . By *S* we denote a *signal* which is considered be a random variable. The basic assumption is that *S* and *N* are *independent*. Our task is to estimate

for some constant *c*. From now on we assume *c*=1.

Let *Z*=1/*S*. Elementary property of variance, shows that

where the second equality follows from **E**[*N*]=0, the third from independence, and the last from .

We need to estimate **E**[*Z*]=**E**[1/*N*]. In general, let *Z*=*f*(*S*) for some well-behaved function (in our case *f*(*S*)=1/*S*). Then, expanding *f*(*S*) in Taylor’s expansion near the mean **E**[*S*] we have

where *S*' is between zero and **E**[*S*]. Taking expected value of the above and noting that the second term is zero we have

(1)

This leads to the following corollary after substituting *f*(*s*)=1/*s* and noting that in this case .

**Corollary 1**  *Assume that* **E***[S]≫1 (large). Then Z=1/S becomes*

*(**2)*

*or even better*

*(**3)*

*as long as the second term above is of smaller order than the first term.*

## VARIANCE OF PURITY MEASUREMENTS

The purity can be expressed as

where represents the area under the first peak and denotes the area under all other peaks. Our goal is to estimate variance of . Observe that

We derive the variance under some simplifying assumptions such as:

(a1) ,

(a2) .

Then we proceed as follows. Denoting and using Corollary Error: Reference source not found we arrive at

(4)

But by (2) of Corollary Error: Reference source not found we have

while by (1) (with ) we also have

We observe that both approximations may be improved by using fuller expansion in (1).

This leads to our first approximation that we formulate as a lemma.

**Lemma 1**  *Under assumptions (a1) and (a2), the following holds*

*(**5)*

*where the approximation depends on how large is.*

We now improve our approximation by dropping assumption (a1) and only postulate (a2). In this case, we need to use a better approximation of **E**[1/*S*].

We write

where . We will assume and are independent.

Throughout this derivation we use the two-term approximation (1) instead of (2). We shall also use

and

Then applying several times (1) we arrive at

(6)

Now we need to approximate and . For the former we use the simple approximation (2) to arrive at

For we use the two term approximation (1) that leads to

Putting everything together into (6) we finally obtain our next approximation

**Lemma 2**  *Under assumption (a2) and proved and are independent, we find*

*(**7)*

*If (a1) holds, that is , then above simplifies to (5) of Lemma Error: Reference source not found*


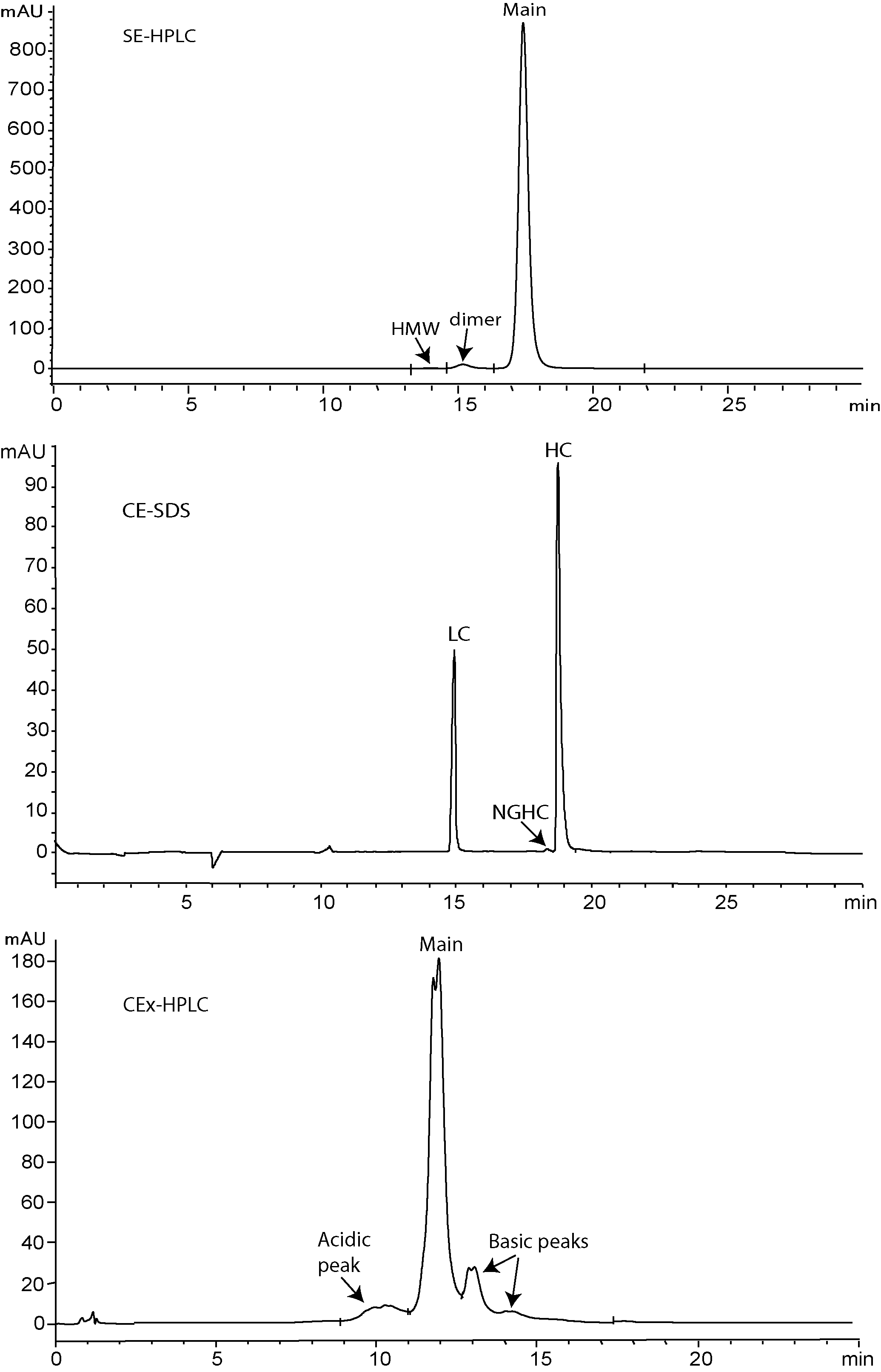


Figure S1. Examples of chromatograms and electrophoregram for mAb: A- SE-HPLC method, B- CE-SDS method, C- CEx-HPLC method (split peak reflect structural isoforms of IgG2 ^30^).


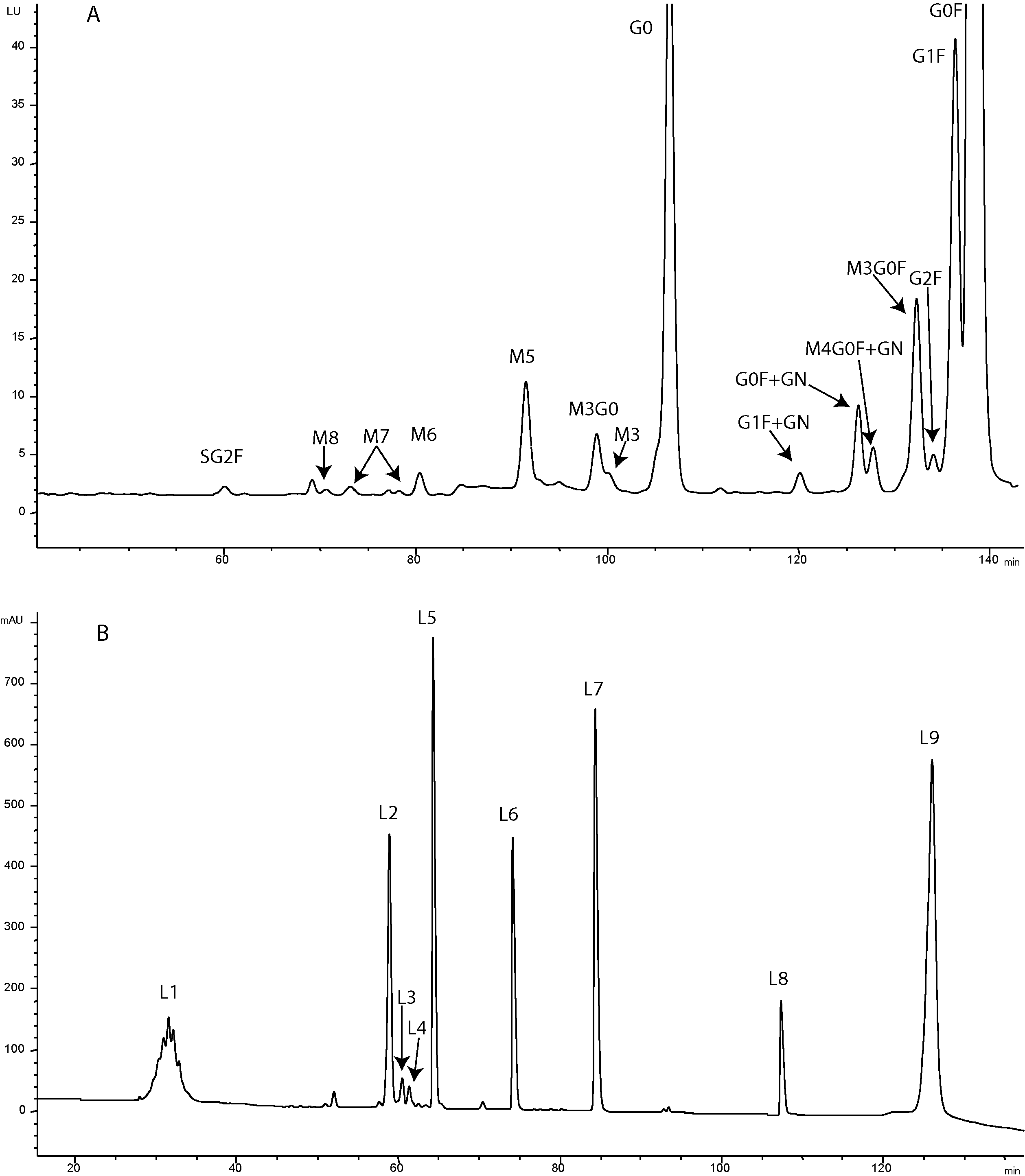


Figure S2. Examples of glycan (**a**) and peptide (**b**) maps.

Figure S3. (**a**) Example chromatogram (hypothetical separation); (**b**) illustration of the rectangle rule ; (**c**) illustration of noise introducing integration bias.

Figure S4. Blending acidic form to create calibration curve for QL calculation.

Table S1.Statistic evaluation of performance characteristics for SE-HPLC, CEx-HPLC, and rCE-SDS methods. The analysis includes: mean, media, 90^th^ percentile, smallest and largest vale for each performance characteristic, n indicates number of available data sets used in the analysis.

(**a**) SE-HPLC methods applied to two proteins modalities (E. coli expressed Fc-Fusion Protein (FcFP) and monoclonal antibody (mAb).

| Performance characteristics: | Parameter and units | Mean | Median | 90^th^ percentile | Smallest | Largest | n |
| --- | --- | --- | --- | --- | --- | --- | --- |
| Specificity | Carryover (% of nominal load) | 0.1 | 0 | 0.1 | 0 | 1.0 | 15 |
|  | % Recovery | 96.2 | 96.4 | 102.2 | 84.2 | 105.3 | 15 |
| Linearity | R^2^ of total peak area vs. conc. (load linearity) | 0.9973 | 0.9994 | 0.9998 | 0.9903 | 0.9998 | 15 |
|  | R^2^ of dimer peak area vs. relative content(minor peak linearity) | 0.9971 | 0.9985 | 0.9996 | 0.9910 | 0.9998 | 14 |
| Repeatability | % RSD for main peak | 0.03 | 0.02 | 0.08 | 0.005 | 0.10 | 20 |
|  | % RSD for dimer | 3.9 | 2.1 | 10.6 | 0.4 | 16.3 | 20 |
| Intermediate Precision | % RSD for main peak | 0.05 | 0.04 | 0.10 | 0.003 | 0.12 | 15 |
|  | % RSD for dimer | 3.7 | 3.3 | 6.3 | 1.5 | 7.1 | 15 |
| Accuracy | % accuracy for main peak | 100.1 | 100.0 | 100.1 | 100.0 | 100.2 | 15 |
|  | % accuracy for dimer | 100.6 | 100.3 | 103.6 | 96.1 | 104.3 | 14 |
| Range | The highest Load (μg) | 320 | 450 | 450 | 105 | 505 | 13 |
|  | The lowest load (μg) | 99 | 101 | 150 | 35 | 150 | 13 |
| Quantitation Limit | QL for dimer (% purity) | 0.2 | 0.1 | 0.3 | 0.02 | 0.3 | 14 |
| Detection Limit | Not reported |  |  |  |  |  |  |

(**b**) CEx-HPLC methods applied to two protein modalities (FcFP and mAb)

| Performance characteristics: | Parameter and units | Mean | Median | 90^th^ percentile | Smallest | Largest | n |
| --- | --- | --- | --- | --- | --- | --- | --- |
| Specificity | Carryover ( % of nominal load) | 0.01 | 0.00 | 0.02 | 0.00 | 0.05 | 13 |
|  | % Recovery | 98.61 | 94.00 | 109 | 87.7 | 122.2 | 13 |
| Linearity | R^2^ of total peak area vs. conc. | 0.9935 | 0.9953 | 0.9987 | 0.9765 | 0.9999 | 14 |
|  | R^2^ of acidic peak area vs. relative content | 0.9936 | 0.9970 | 0.9995 | 0.9660 | 0.9998 | 13 |
|  | R^2^ of basic peak area vs. relative content. | 0.9796 | 0.9960 | 0.9980 | 0.8300 | 0.9993 | 11 |
| Repeatability | % RSD for main peak | 0.5 | 0.4 | 1.0 | 0.1 | 2.4 | 19 |
|  | % RSD for acidic peak | 1.6 | 1.3 | 2.9 | 0.1 | 5.9 | 19 |
|  | % RSD for basic peak | 2.5 | 1.8 | 4.8 | 0.3 | 7.2 | 17 |
| Intermediate Precision | % RSD for the main peak | 0.9 | 0.7 | 1.4 | 0.2 | 2.6 | 14 |
|  | % RSD for acidic peak | 4.1 | 2.7 | 6.9 | 0.3 | 23.6 | 14 |
|  | % RSD for basic peak | 12.6 | 5.9 | 29.4 | 0.6 | 34.6 | 12 |
| Accuracy | % accuracy for main peak | 100.1 | 100.3 | 100.7 | 99.0 | 100.9 | 14 |
|  | % accuracy for acidic | 102.2 | 101.3 | 109.8 | 93.3 | 110.5 | 13 |
|  | % accuracy for basic | 96.2 | 96.8 | 100.0 | 84.2 | 108.1 | 11 |
| Range | The highest conc. (mg/ml) | 3.7 | 3.0 | 5.0 | 0.5 | 10.5 | 13 |
|  | The lowest conc. (mg/ml) | 1.5 | 1.0 | 3.1 | 0.2 | 5.1 | 13 |
| Quantitation Limit | QL for acidic (% purity) | 0.7 | 0.5 | 1.2 | 0.1 | 2.0 | 13 |
|  | QL for basic (% purity) | 0.6 | 0.5 | 0.8 | 0.2 | 2.1 | 12 |
| Detection Limit | Not reported |  |  |  |  |  |  |

(**c**) rCE-SDS method applied to mAbs

| Performance characteristics: |  | Mean (%) | Median | 90^th^ percentile | Smallest | Largest | n |
| --- | --- | --- | --- | --- | --- | --- | --- |
| Specificity | Carryover | 0.00 | 0.00 | 0.00 | 0.00 | 0.00 | 5 |
| Load Linearity | R^2^ of total peak area vs. conc. | 0.9932 | 0.9922 | 0.9978 | 0.9899 | 0.9983 | 9 |
| Linearity of Minor Peak | R^2^ of NGHC | 0.9898 | 0.9950 | 0.9987 | 0.9769 | 0.9993 | 5 |
| Precision-Repeatability | % RSD for HC | 0.36 | 0.37 | 0.68 | 0.06 | 0.80 | 15 |
|  | % RSD for LC | 0.48 | 0.35 | 0.72 | 0.20 | 0.94 | 9 |
|  | % RSD for NGHC | 4.81 | 4.87 | 7.64 | 0.90 | 8.60 | 4 |
| Precision-Intermediate Precision | % RSD for HC | 0.87 | 0.76 | 1.40 | 0.27 | 2.10 | 11 |
|  | % RSD for LC | 1.77 | 1.62 | 2.77 | 0.66 | 4.30 | 10 |
|  | % RSD for NGHC | 5.99 | 7.15 | 8.54 | 0.90 | 8.78 | 4 |
| Accuracy-%Main Peak | % accuracy for LC | 99.94 | 100.10 | 100.50 | 98.80 | 100.59 | 9 |
|  | % accuracy for HC | 102.22 | 99.90 | 104.88 | 99.00 | 122.00 | 9 |
|  | % accuracy for NGHC | 101.05 | 101.27 | 107.13 | 93.20 | 107.46 | 6 |
| Range | The highest conc. (mg/ml) | 1.51 | 1.50 | 2.00 | 0.75 | 2.00 | 11 |
|  | The lowest conc. (mg/ml) | 0.48 | 0.50 | 0.50 | 0.25 | 0.50 | 11 |
| Quantitation Limit | QL for NGHC (%) | 0.19 | 0.13 | 0.41 | - | 0.48 | 10 |
| Detection Limit | Not reported |  |  |  |  |  |  |

Table S2. Design of experiment for F-test.

| Sample | # of replicates on day 1 | # of replicates on day 2 | # of analytes (peaks) | Acquisition rates |
| --- | --- | --- | --- | --- |
| Peptide map | 3 | 3 | 9 | 1, 5, and 20 Hz |
| Glycan map | 3 | 9 | 16 | 2.5 Hz |

Table S3. Design of experiment for testing UBCI

| Part | Method | # of replicates | # of analytes (# peaks) | # of protein analyzed | Acquisition rate [Hz] |
| --- | --- | --- | --- | --- | --- |
| a | Peptide map | 3 | 9 | 1 | 0.25, 1, 5, and 20 |
|  | Glycan map | 3, and 9 | 16 | 1 | 2.5 |
| b | SE-HPLC | 3 and 40 | 2 or 3 | 6 | 2, and 2.5 |
|  | CEx-HPLC | 3 | 3 | 3 | 2 |

Table S4. Parameters of the regression form blending experiment used to calculate static QL using equation 18

|  | Slope | STD | QL |
| --- | --- | --- | --- |
| Standard Deviation of residuals of the regression line | 0.9617 | 34.69 | 4.7 % |
| Standard deviation (standard error) of Y- Intercept |  | 20.96 | 2.8 % |

Table S5.Calculation of dynamic QL, based on equation 19

|  | ASTM noise  [mAU] | Peak height  [mAU] | Purity  [%] | QL  [%] |
| --- | --- | --- | --- | --- |
| Inj-1 | 0.0401 | 4.4852 | 5.0 | 0.449 |
| Inj-2 | 0.0583 | 4.3246 | 4.8 | 0.648 |
| Inj-3 | 0.0523 | 4.4483 | 5.1 | 0.598 |
|  |  |  | Average | **0.565** |
